# Shiga Toxin 1–Producing *Shigella sonnei* Infections, California, United States, 2014–2015

**DOI:** 10.3201/eid2204.151825

**Published:** 2016-04

**Authors:** Katherine Lamba, Jennifer A. Nelson, Akiko C. Kimura, Alyssa Poe, Joan Collins, Annie S. Kao, Laura Cruz, Gregory Inami, Julie Vaishampayan, Alvaro Garza, Vishnu Chaturvedi, Duc J. Vugia

**Affiliations:** California Department of Public Health, Richmond, California, USA (K. Lamba, A.C. Kimura, A. Poe, L. Cruz, G. Inami, V. Chaturvedi, D.J. Vugia);; County of San Diego Health and Human Services Agency, San Diego, California, USA (J.A. Nelson, A.S. Kao);; San Joaquin County Public Health Services, Stockton, California, USA (J. Collins, J. Vaishampayan, A. Garza)

**Keywords:** Shigella, sonnei, dysenteriae, flexneri, Escherichia coli, Shiga toxin, shigellemia, STEC, Stx-1 shigellosis, bacteria, diarrhea, cytotoxins, enteric infections, gastrointestinal, travel, antimicrobial resistance, antibiotic resistance, San Joaquin County, County of San Diego, California, United States, Mexico

## Abstract

Shiga toxins (Stx) are primarily associated with Shiga toxin–producing *Escherichia coli* and *Shigella dysenteriae* serotype 1. Stx production by other shigellae is uncommon, but in 2014, Stx1-producing *S. sonnei* infections were detected in California. Surveillance was enhanced to test *S. sonnei* isolates for the presence and expression of *stx* genes, perform DNA subtyping, describe clinical and epidemiologic characteristics of case-patients, and investigate for sources of infection. During June 2014–April 2015, we identified 56 cases of Stx1-producing *S. sonnei*, in 2 clusters. All isolates encoded *stx_1_* and produced active Stx1. Multiple pulsed-field gel electrophoresis patterns were identified. Bloody diarrhea was reported by 71% of case-patients; none had hemolytic uremic syndrome. Some initial cases were epidemiologically linked to travel to Mexico, but subsequent infections were transmitted domestically. Continued surveillance of Stx1-producing *S. sonnei* in California is necessary to characterize its features and plan for reduction of its spread in the United States.

Shiga toxins (Stxs) are cytotoxins that mediate severe gastrointestinal disease caused by Shiga toxin–producing *Escherichia coli* (STEC) and *Shigella dysenteriae* serotype 1 (*1*). *S. dysenteriae* 1 produces the prototype Shiga toxin (Stx), and STEC can produce 2 groups of Stxs: Stx1 and Stx2. Vascular damage caused by Stxs in the colon, kidneys, and central nervous system may result in hemorrhagic colitis, or more severe conditions such as hemolytic uremic syndrome (HUS) (*2*). Stx2 has been shown to be more virulent than Stx1 (*3*), and adverse clinical outcomes such as HUS are more frequently associated with Stx2-producing strains of STEC than Stx1-producing strains (*4*). Antimicrobial drug treatment for STEC infections and late or inappropriate antimicrobial drug treatment for *S. dysenteriae* 1 infections have been associated with an increased risk for HUS (*5*,*6*).

Although Shiga toxins have been associated with STEC and *S. dysenteriae* 1, infections caused by other types of Stx-producing *Shigella* spp. have been recognized in recent years. Sporadic infections with Stx1-producing *S. dysenteriae* serotype 4 and *S. flexneri* in persons with a history of travel to the Caribbean island of Hispañiola have been characterized in the United States and Canada (*7*–*9*). A recent survey of *Shigella* isolates from persons with a history of travel to the Caribbean found that 21% of isolates encoded and produced Stx; positive strains were *S. flexneri* 2a, *S. flexneri* Y, and *S. dysenteriae* 4 (*10*). The same Stx-converting bacteriophage was identified among these isolates, suggesting the emergence of Stx-producing shigellae in this region was caused by spread of the phage to multiple *Shigella* species and serotypes. One case of infection with Stx1-producing *S. sonnei* in a patient from Germany who had a history of travel to Ukraine (*11*) and one instance of isolation of *stx_2a_-*encoding *S. sonnei* from a patient from Finland who had a history of travel to Morocco have been described (*12*). Although these novel strains of Stx-producing shigellae have been reported recently, data regarding the clinical characteristics and epidemiology of these infections remain limited.

In the United States, infections with Stx-producing organisms are primarily caused by STEC; *S. dysenteriae* 1 infections are rare. Of laboratory-confirmed cases of shigellosis reported in the United States, ≈75% are caused by *S. sonnei* (*13*). *S. sonnei* infections are typically less severe than infections with *S. dysenteriae* 1 and are characterized by diarrhea, which may be bloody and accompanied by fever, nausea, and abdominal cramps. Illnesses are usually self-limited and resolve within 5–7 days of onset. Extraintestinal complications such as bacteremia and urogenital infections are rare but have been documented (*14*–*17*). Although antimicrobial drug treatment is generally unnecessary for patients with uncomplicated *S. sonnei* infections, antimicrobial drugs are often used to limit the duration of illness and communicability and to reduce illness severity (*18*).

In August 2014, Stx1-positive fecal samples from 2 patients tested by using enzyme immunoassay were reported to the County of San Diego Health and Human Services Agency (COSD HHSA; San Diego, CA, USA); the same clinical specimens showed positive *S. sonnei* culture results. COSD HHSA initiated an investigation and notified the California Department of Public Health (CDPH). Surveillance was enhanced retrospectively and prospectively in California to confirm Stx-producing *S. sonnei* isolates, identify additional cases, describe the clinical and epidemiologic characteristics of infected persons, monitor for severe clinical outcomes such as HUS, and to investigate for potential sources of infection. We subsequently identified 2 clusters of Stx-producing *S. sonnei*; the second cluster continued into late 2015. We report our findings on the initial 56 confirmed cases of Stx1-producing *S. sonnei* identified during June 2014–April 2015.

## Methods

### Case Finding

We defined a suspected case as detection of *S. sonnei* and Stx or *stx* genes in a clinical specimen collected during June 2014–April 2015 from a resident of California. We implemented several methods to identify suspected cases for laboratory confirmation and epidemiologic investigation. CDPH notified California local health departments of unusual detections and requested that suspected cases be reported. In addition, regular reviews of shigellosis and STEC electronic surveillance data were conducted to identify suspected cases. Some local health departments communicated with healthcare providers in their administrative areas to request reporting of suspected cases. After the detection of a cluster of suspected cases in San Joaquin County in February 2015, San Joaquin County Public Health Services prospectively enhanced laboratory surveillance and requested that local laboratories submit all *Shigella* isolates for Stx testing. Stx-positive broths and *S. sonnei* isolates for all suspected cases were forwarded to the CDPH Microbial Diseases Laboratory to confirm the presence and expression of *stx* genes in *S. sonnei*.

### Laboratory Testing

All isolates from patients with suspected cases were identified by using a commercial rapid identification system (VITEK 2 Compact, bioMérieux, Bio Marcy l’Etoile, France) or *Shigella* antisera. Isolates were further characterized by using a multiplex real-time PCR for *stx_1_* and *stx_2_* genes according to a scheme for STEC testing described by Probert et al (*19*). The Vero cell neutralization assay was used to detect active Stx. Pulsed-field gel electrophoresis (PFGE) was performed by using PulseNet protocols (*20*).

### Case Definitions

We defined a confirmed case as isolation of *S*. *sonnei* positive for *stx* genes and active Stx production from a clinical specimen collected during June 2014–April 2015 from a resident of California. A secondary case was defined as a confirmed case in a person with illness onset >12 hours but within 2 weeks after household or other close contact with a person with a diarrheal illness. Cases in persons for whom data were available regarding ill contacts that did not meet the secondary case criteria were considered primary cases. Cases of undiagnosed diarrheal illness in contacts of suspected or confirmed case-patients were excluded from the investigation.

### Data Collection

After receiving a report of Stx-positive feces/STEC/HUS or a diagnosis of shigellosis in a patient from a healthcare provider or laboratory, California local health department personnel routinely interview patients by using a standardized shigellosis or STEC/HUS case report form. We re-interviewed patients who had a suspected case of Stx-producing *S. sonnei* with a standardized investigation questionnaire that collected information on demographics, symptoms, treatment, clinical outcomes, risk factors for shigellosis, and exposure history. Suspected case-patients were queried regarding exposures during the 7 days before symptom onset. We extracted data from the local health department standardized shigellosis or STEC case report form if case-patients were not reached for re-interview with the investigation questionnaire and retrieved clinical data from medical records for case-patients lost to follow-up.

### Data Analysis

Illness onset date was unavailable for 1 case-patient and was estimated as the date 3 days before specimen collection. For case-patients symptomatic at the time of the last interview, we calculated duration of symptoms by subtracting illness onset date from interview date. Data were summarized with descriptive statistics; we used the χ^2^ test, Fisher’s exact test, and Student *t*-test to compare characteristics of case-patients in clusters 1 and 2 and by age category (<5 vs. >5 years of age). We used the binomial test to compare the number of Hispanic case-patients in each cluster to population proportions based on 2014 American Community Survey data (*21*). A p value of <0.05 was considered significant. We used SAS version 9.4 (SAS Institute, Inc., Cary, NC, USA) for analyses.

## Results

**Figure 1:**
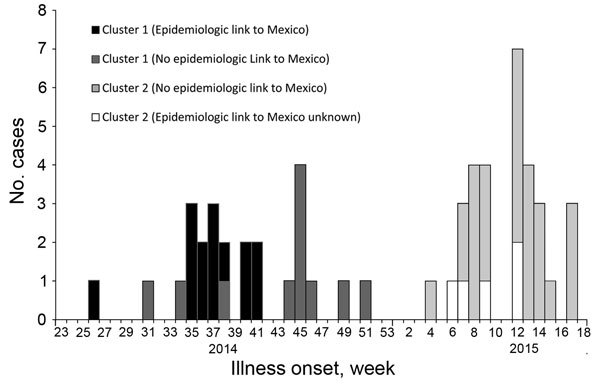
Epidemic curve of Shiga toxin 1–producing *Shigella sonnei* cases in California (N = 56), by week of illness onset, cluster, and epidemiologic link to Mexico, June 2014–April 2015. Cluster 1, southern California, June–December 2014 (n = 25); cluster 2, San Joaquin County, northern California, January–April 2015 (n = 31). Illness onset week designated using Centers for Disease Control and Prevention Morbidity and Mortality Weekly Report weeks.

During June 2014–April 2015, we identified a total of 56 cases of Stx1-producing *S. sonnei* in 2 epidemiologically distinct clusters in California. Cluster 1 consisted of 25 cases in persons epidemiologically linked to southern California with illness onset dates during June 26–December 18, 2014 (Figure 1). Cluster 2 consisted of 31 cases in residents of San Joaquin County in northern California with illness onset dates during January 28–April 28, 2015. The median age of all case-patients was 19.5 years (range 1–71 years); 54% were male. Eleven (20%) case-patients were children <5 years of age.

### Laboratory Testing

**Figure 2:**
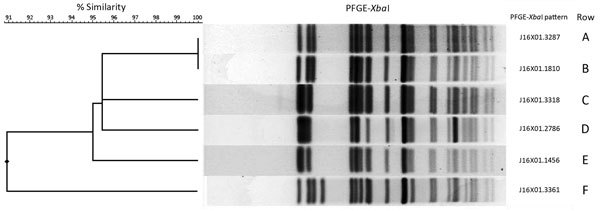
Dendrogram and selected pulsed-field gel electrophoresis (PFGE) patterns of *Xba*I-digested Shiga toxin 1–producing *Shigella sonnei* isolates from California. Predominant *Xba*I patterns identified in cluster 1 are shown in rows A and B and outlier on row F. Predominant patterns identified in cluster 2 are shown in rows C and E and outlier on row D. ID no., identification number.

The 56 isolates tested were confirmed to be *S. sonnei* by the rapid identification system or by agglutination in group D antiserum. All isolates tested by the multiplex PCR were positive for the *stx_1_* and negative for the *stx_2_* gene and showed active Stx production by neutralization with Stx1 antiserum in the Vero cell assay. We constructed a dendrogram of predominant and selected PFGE patterns after *Xba*I digestion (Figure 2).

### Clinical Summary

Overall clinical characteristics of the 56 cases (Table 1) did not differ significantly by cluster (data not shown). Case-patients were interviewed by using the investigation questionnaire a median of 67 and 11.5 days, respectively, after illness onset in clusters 1 and 2. Symptoms most commonly reported were diarrhea (100%), abdominal cramps (87%), fever (72%), and bloody diarrhea (71%). Twelve (21%) case-patients were hospitalized and 27 (53%) of 51 sought medical care at an emergency department. Case-patients <5 years of age were more likely to have fever (100% vs. 67%, p = 0.054) and bloody diarrhea (80% vs. 68%; not statistically significant) than were case-patients >5 years of age. The median duration of symptoms was 5 days (interquartile range 3–7 days), and median length of hospitalization was 3 days (interquartile range 1.5–4 days). Six case-patients in cluster 2 were symptomatic at the time of interview; illness duration ranged from 5–12 days. No cases of HUS were identified, and no patients died.

**Table 1:** Clinical characteristics of patients with Shiga toxin 1–producing *Shigella sonnei* infections, California, June 2014–April 2015*

Characteristic	Value
Symptoms	
Diarrhea	56/56 (100.0)
Abdominal cramps	45/52 (86.5)
Fever†	36/50 (72.0)
Bloody diarrhea	36/51 (70.6)
Chills	26/41 (63.4)
Nausea	22/39 (56.4)
Vomiting	21/48 (43.8)
Headache	10/33 (30.3)
Outcomes	
Emergency department visit	27/51 (52.9)
Hospitalization	12/56 (21.4)
Hemolytic uremic syndrome	0/43
Death	0/56
Median duration of symptoms, d (IQR)	5 (3–7)‡
Median length of hospitalization, d (IQR)	3 (1.5–4)§
Antimicrobial resistance	
Trimethoprim/sulfamethoxazole	27/29 (93.1)
Ampicillin	6/25 (24.0)
Ciprofloxacin	0/22
Ceftriaxone	0/13
Treatment	
Received any treatment	48/54 (88.9)
Treated with antimicrobials	43/53 (81.1)
Antimicrobial treatment¶	
Ciprofloxacin	19 (45.2)
Metronidazole	12 (28.6)
Ceftriaxone	5 (11.9)
Levofloxacin	5 (11.9)
Trimethoprim/sulfamethoxazole	4 (9.5)
Azithromycin	4 (9.5)
Ampicillin	3 (7.1)
Amoxicillin	2 (4.8)
Other or unknown	3 (7.1)
Median interval from diarrhea onset to start of antimicrobial treatment, d (IQR)	3 (1–5)#
Treated with intravenous fluids	23/46 (50.0)
Received other treatment**	17/54 (31.5)

*Values are no. patients/no. with information available (%) excepted as indicated. IQR, interquartile range. †Based on self-reported data; 19 (52.8%) of 36 patients had documented temperature of ≥38°C. ‡n = 39; includes 6 patients in cluster 2 still symptomatic at time of interview. Illness duration range 5–12 d. §n = 11. ¶n = 42. Because 10 case-patients were treated with >1 antimicrobial drug, total is >100%. #n = 35. **Antiemetics, pain medication, nonprescription antidiarrheal medication.

*S. sonnei* was isolated from fecal specimens from 54 case-patients, from a blood specimen from 1 case-patient, and from a urine specimen from 1 case-patient. The case-patient who had bacteremia was a woman, 57 years of age, who had a history of substantial underlying medical conditions, including cirrhosis of the liver secondary to hepatitis C; she was hospitalized for hepatic encephalopathy. Her symptoms included abdominal pain and diarrhea, and computed tomography scan results of the abdomen indicated colitis and mesenteric lymphadenopathy; however, fecal culture was negative for *Shigella* and *E. coli* O157. The patient was treated with levofloxacin for her infection and discharged after 6 days. The case-patient from whom *S. sonnei* was isolated from a urine specimen was a 50-year-old woman with a diarrheal illness and clinical urinary tract infection (dysuria, pyuria >10 leukocytes/high power field). Urine culture for this patient also yielded *E. coli* and *Enterococcus faecalis*. *S. sonnei* was not isolated from her fecal sample.

Among the 29 isolates for which antimicrobial drug susceptibility testing results were available from clinical laboratories, we documented several antibiogram profiles. The most commonly identified profile (n = 16, 55%) showed resistance to trimethoprim/sulfamethoxazole and susceptibility to ciprofloxacin and ampicillin. Four (14%) isolates were resistant to trimethoprim/sulfamethoxazole and ampicillin but susceptible to ciprofloxacin. Of 10 isolates from case-patients in cluster 2, six (60%) were resistant to ampicillin; none were resistant in cluster 1. 

Of 53 case-patients with data available, 43 (81%) were treated with antimicrobial drugs. Most (n = 33, 77%) case-patients treated with antimicrobial drugs were treated with 1 drug, most frequently with ciprofloxacin (13/32, 41%). Among the case-patients treated with >1 antimicrobial drug, ciprofloxacin and metronidazole was the most common combination (6/10, 60%). A 4-year-old case-patient was treated with trimethoprim/sulfamethoxazole, to which the isolate was resistant. This patient’s treatment was started 5 days after diarrhea onset, and no severe outcomes were observed. The median number of days from diarrhea onset to start of antimicrobial treatment was 3 days (interquartile range 1–5 days), and 23 (66%) of 35 case-patients began antimicrobial treatment within 4 days of diarrhea onset. No differences were identified between case-patients <5 versus >5 years of age with respect to timing of antimicrobial drug treatment initiation or proportion treated with antimicrobial drugs.

### Epidemiologic Summary

#### Cluster 1

In this cluster, 24 of the 25 case-patients were residents of San Diego (n = 20), Orange (n = 2), and Riverside (n = 2) counties in southern California. One case-patient from San Joaquin County had illness onset in June 2014 after household contact with a visitor from southern California who had an undiagnosed diarrheal illness. We included this case-patient in cluster 1 on the basis of the illness onset date, contact with the ill southern California visitor, and positive test results for an isolate with the PFGE *Xba*I pattern predominant in cluster 1. 

The median age of case-patients in this cluster was 10 years (range 2–64 years; Table 2). Case-patients were more likely to be Hispanic than the general population in those 4 counties (60.0% vs. 34.7%; p = 0.0085). Nine were considered primary case-patients and 16 were categorized as secondary; of the secondary case-patients, 7 had been exposed to another confirmed case-patient, and 9 to a contact with an undiagnosed diarrheal illness (Table 2). We identified 4 clusters of household transmission involving >2 confirmed cases, and a range of 2–5 cases of diarrheal illness in each household. 

**Table 2:** Epidemiologic characteristics of Shiga toxin 1–producing *Shigella sonnei* cases by cluster, California, June 2014–April 2015

Characteristic	No. (%)	
	Cluster 1, n = 25	Cluster 2, n = 31
Male sex	12 (48.0)	18 (58.1)
Age, y		
0–4	6 (24.0)	5 (16.1)
5–9	6 (24.0)	0
10–19	5 (20.0)	6 (19.4)
20–29	1 (4.0)	3 (9.7)
30–39	4 (16.0)	3 (9.7)
40–49	0	2 (6.5)
50–59	2 (8.0)	8 (25.8)
≥60	1 (4.0)	4 (12.9)
Ethnicity		
Hispanic	15 (60.0)	14 (45.2)
Non-Hispanic	10 (40.0)	12 (38.7)
Unknown	0	5 (16.1)
Case status		
Primary case	9 (36.0)	18 (64.3)*
Secondary case	16 (64.0)	10 (35.7)*
Epidemiologic risk factors		
Epidemiologic link to Mexico	14 (56.0)	0
Homeless	0	4 (12.9)
Illicit drug use	NA†	7 (22.6)
Men who have sex with men‡	0	0

*n = 28. †Not available, data not collected in Cluster 1 investigation. ‡Denominator is men ≥18 y of age with data available: n = 2 for cluster 1 and n = 8 for cluster 2.

Five of 9 primary case-patients reported travel to Mexico during their exposure period, and 9 of 16 secondary case-patients were exposed to someone with a diarrheal illness who lived in or visited Mexico (Table 2). Overall, 14 (56%) cases were epidemiologically linked to Mexico, primarily Baja California. None of the case-patients whose illness onset dates were after mid-October 2014 (n = 8) reported epidemiologic links to Mexico. Of the 5 primary case-patients with a travel history to Mexico, none were there for all of the 7 days before illness onset. No common point sources of transmission were identified in Mexico or California. 

The predominant PFGE *Xba*I pattern for isolates from patients in this cluster was J16X01.3287, identified in 12 (48%) isolates, including the San Joaquin County case-patient isolate. J16X01.1810 was the second most common pattern, identified in 4 (16%) isolates; 8 additional PFGE *Xba*I patterns were identified. We observed no association between PFGE patterns and epidemiologic links to Mexico.

#### Cluster 2

The 31 case-patients in this cluster were residents of San Joaquin County. Their median age was 33 years (range 1–71 years; Table 2), which was substantially older than that for cluster 1 (p = 0.0035). Eighteen cases were categorized as primary and 10 as secondary; 8 were secondary to another confirmed case. A smaller proportion of case-patients in cluster 2 were categorized as secondary than in cluster 1 (35.7% vs. 64.0%; p = 0.0398). 

We identified 4 clusters of household transmission involving >2 confirmed cases in this cluster; a range of 2–4 cases of diarrheal illness were reported in each household. None of the case-patients reported a history of travel outside of California or close contact with anyone who lived in or visited Mexico. Case-patients in cluster 2 were significantly less likely to have an epidemiologic link to Mexico than those in cluster 1 (0% vs. 56.0%; p<0.0001). Four case-patients were homeless and 7 reported illicit drug use. 

We identified no common point sources of transmission for this cluster. Aside from the identification of 1 case-patient in San Joaquin County in cluster 1, which occurred 7 months before cluster 2, no epidemiologic connection to cluster 1 was established. PFGE *Xba*I pattern J16X01.1456 was identified in 22 (71%) cluster 2 isolates, but no common source was identified among these cases. One PFGE *Xba*I pattern, J16X01.3318, was common to both clusters, identified in 2 cases from cluster 1 and 7 (23%) cases from cluster 2; however, no epidemiologic connection was found among the case-patients. Two additional PFGE *Xba*I patterns were identified in cluster 2 isolates.

## Discussion

We documented 56 cases of Stx-producing *S. sonnei* in the United States in 2 clusters, beginning in June 2014. All 56 *S. sonnei* isolates were confirmed to encode *stx_1_* and produce active Stx1. The clinical presentation of case-patients appeared typical for illness caused by *S. sonnei,* but more patients reported bloody diarrhea than expected. Although several initial cases detected in southern California had epidemiologic links to Mexico, most cases appeared to result from sustained domestic transmission, suggesting this pathogen may become more prevalent in California and in the United States.

Overall, the patients’ diarrheal illnesses appeared typical for shigellosis caused by *S. sonnei*, and no cases of HUS were identified. The proportion of patients reporting bloody diarrhea, 71%, was higher than expected, but it is unclear whether the presence of Stx1 increased the risk for bloody diarrhea in these case-patients. A study of children with shigellosis in Bangladesh reported that those infected with *S. dysenteriae* type 1 had more grossly bloody feces than did those infected with other *Shigella* species (78% vs. 33%), likely caused by Stx (*22*). In our study, the case-patient with bacteremia had several underlying medical conditions including liver cirrhosis, a condition previously associated with shigellemia (*23*). Further studies are needed regarding the effects of Stx1 production in the pathogenesis and virulence of *S. sonnei* and the risk factors associated with developing adverse outcomes atypical for *S. sonnei* infections.

In our investigation, most case-patients received antimicrobial drug treatment, and no adverse outcomes or cases of HUS were observed. Antimicrobial drug therapy is widely used for treatment of patients with shigellosis, and early and effective treatment in patients infected with *S. dysenteriae* 1 has been shown to decrease the risk for HUS (*24*). However, delayed treatment or treatment with antimicrobial drugs to which the *S. dysenteriae* 1 isolate is resistant may be associated with an increased risk for HUS (*3*). The mechanisms by which antimicrobial treatment may precipitate *S. dysenteriae* 1–associated HUS are not well understood. Antimicrobial drug treatment for STEC infections is hypothesized to promote Stx production, and consequently increase the risk of developing HUS, so it is generally not recommended (*6*,*25*). The effects of antimicrobial drug treatment on pathogenesis or Stx production of Stx1-producing *S. sonnei* infections remain unclear. We identified multiple resistance patterns among the isolates in this study, suggesting that etiologic strains are heterogeneous. Considering the increasing prevalence of drug-resistant *Shigella* in the United States (*26*), clinicians should be aware of resistance patterns and monitor for potential adverse outcomes if deciding to treat Stx1-producing *S. sonnei* infections with antimicrobial drugs.

Our clinical data are subject to limitations. We excluded undiagnosed cases of diarrheal illness in contacts of confirmed case-patients from our investigation. Some of these persons might have had clinically mild infections with Stx1-producing *S. sonnei*. However, the possibility of missing adverse clinical outcomes in some patients is small, and because HUS is a reportable condition in California, unrecognized cases of HUS are unlikely. The lag between illness onset and case-patient re-interview in cluster 1 was unlikely to bias results because most pertinent data were collected during the initial standard shigellosis or STEC case investigation. The lag in the second interviews confirmed no adverse outcomes in case-patients.

Our investigation of 2 epidemiologically distinct clusters of Stx-producing *S. sonnei* infections suggests that emerging strains of *S. sonnei* may have initially been introduced into southern California by travelers returning from Mexico but are now circulating domestically. Stx1-producing *S. sonnei* strains have not been reported in Mexico, but our investigation suggests that they may be circulating in the Baja California region. 

No clear connections between the clusters in southern and northern California were established. The introductory sources for cluster 2 remain unknown, but possibilities include unrecognized spread from southern California, new unrecognized introductions by travelers returning from abroad, or conversion of a naive strain of *S. sonnei* by an Stx1-converting bacteriophage and subsequent spread. The lack of an epidemiologic link to international travel among cases in cluster 2 and the substantial number of secondary cases caused by person-to-person transmission in both clusters demonstrate the potential for continued domestic transmission and spread in the United States.

The number of cases reported in this study is likely an underestimate of the true number of infections caused by Stx1-producing *S. sonnei* in California. Additional cases have likely gone unrecognized because persons with mild infections did not seek medical care or because laboratories and healthcare providers did not identify or report specimens positive for both Stx and *S. sonnei*. Routine Stx testing and fecal culture for patients with a clinically compatible illness may increase detection of infections with this pathogen in the future. Prompt identification is reliant upon timely reporting of results of Stx tests and fecal culture. The increasing use of multipanel culture-independent diagnostic tests may aid detection of Stx-producing *Shigella* infections. However, results must be interpreted with caution, and confirmatory testing by culture will be needed to distinguish between infection with Stx-producing *Shigella* and co-infections with *Shigella* and STEC. Enhanced surveillance by public health officials is advised, particularly for the possibility of emergence and spread of Stx2-producing *Shigella* infections, which may be more clinically severe. Transmission in high-risk settings such as childcare is of particular concern because of the potential for severe clinical outcomes in the affected populations. Although severe outcomes were not observed in the patient infected with *stx_2a_-*encoding *S. sonnei* reported by Nyholm et al. (*12*), the potential for increased virulence of Stx-producing *S. sonnei* cannot be ruled out at this time.

Challenges associated with surveillance, reporting, clinical management, and public health follow-up of both shigellosis and STEC in the United States may become more prevalent as the incidence of Stx-producing *Shigella* infections increases. Because Stx-producing *Shigella* infections have been identified in Europe and the Americas, clinicians and public health officials worldwide should remain vigilant for cases of HUS or other sequelae in infected persons. Since our investigation concluded, cases of Stx1-producing *S. sonnei* have continued to be identified and reported from San Joaquin County. Surveillance for additional cases is ongoing in California to better characterize the clinical and epidemiologic features of infections with Stx-producing *S. sonnei*.
